# Supplementary material for: α-Terpineol Induces Shelterin Components TRF1 and TRF2 to Mitigate Senescence and Telomere Integrity Loss via A Telomerase-Independent Pathway
Source: Antioxidants (Basel). 2024 Oct 17;13(10):1258. doi: 10.3390/antiox13101258 (PMC11504354; doi:10.3390/antiox13101258)
Supplement: Supplementary file 1 [file antioxidants-13-01258-s001.zip › antioxidants-3238673-supplementary.pdf]

Supplementary data

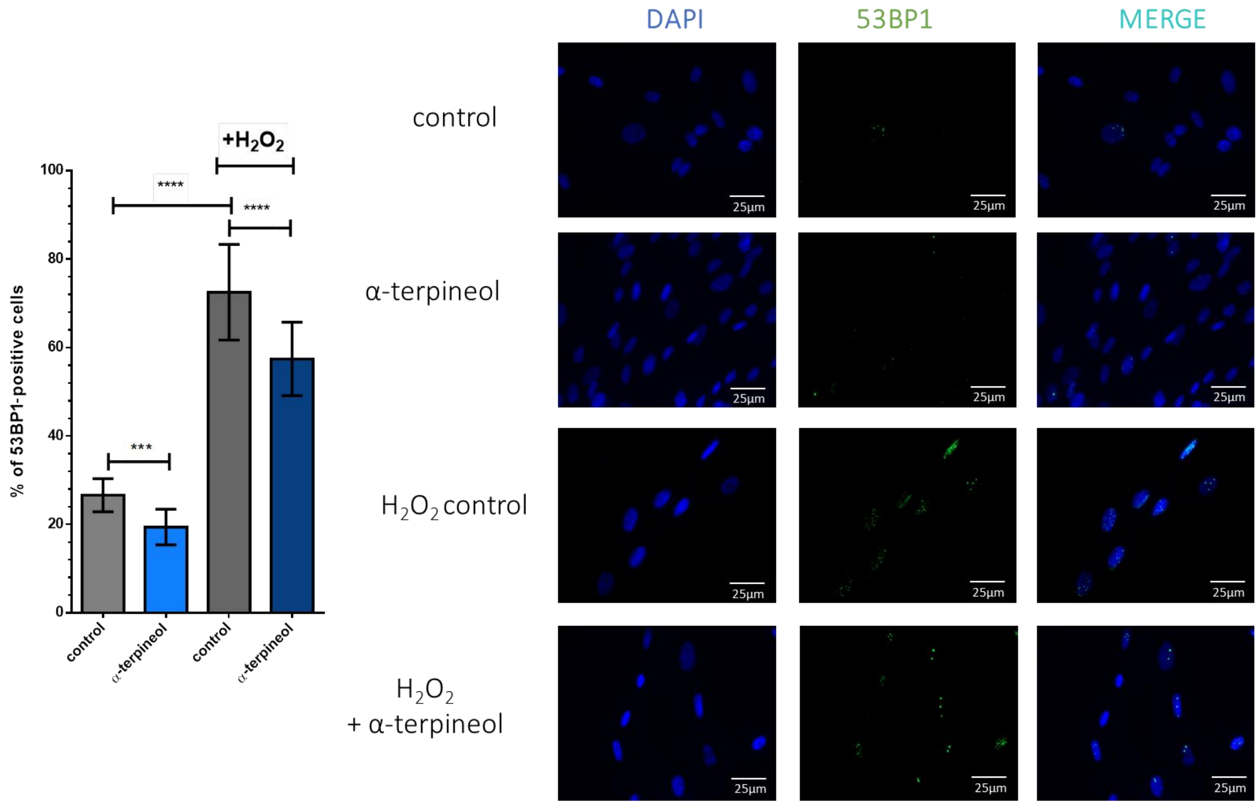

Figure S1. Treatment with terpineol reduces oxidative stress induced DNA damage and enhances cellular resistance**.** A) Number of fibroblasts treated with terpineol or DMSO (solvent control) for 24 h following treatment with 300 μM H_2O_2 for 2.5 h and a five day recovery period. Quantification of 53BP in the nuclei and representative images of human fibroblasts treated *α*-terpineol or DMSO (solvent control) for 24 h following treatment with 300 μM H_2O_2 for 2.5 h. 53BP was detected using an anti-53BP (green) antibody. DNA was co-stained with DAPI (4′, 6′-diamidino-2-phenylindole). Representative images of nucleus and 53BP are shown with or without H_2O_2 treatment. ****: $p < 0.0001$, ***: $p < 0.001$.
